# The efficacy of botulinum toxin a injections in pelvic floor muscles in chronic pelvic pain patients: a systematic review and meta-analysis

**DOI:** 10.1007/s00192-022-05115-7

**Published:** 2022-04-01

**Authors:** Melle A. Spruijt, Wenche M. Klerkx, Johannes C. Kelder, Kirsten B. Kluivers, Manon H. Kerkhof

**Affiliations:** 1 Department of Obstetrics and Gynaecology, St. Antonius Hospital, Koekoekslaan 1, 3435 CM Nieuwegein, The Netherlands; 2 Department of Obstetrics and Gynaecology, Radboud University Medical Center, Nijmegen, The Netherlands; 3 Department of Epidemiology, St. Antonius Hospital, Nieuwegein, The Netherlands; 4 Department of Gynaecology and Reconstructive pelvic surgery, Curilion Women’s Health Clinic, Haarlem, The Netherlands

**Keywords:** Chronic pain, Botulinum toxin A, Pelvic floor, Pelvic pain, Intramuscular injection

## Abstract

**Introduction and hypothesis:**

Chronic pelvic pain (CPP) is a common multifactorial condition affecting 6 to 27% of women aged 18–50 years worldwide. This study was conducted to review and meta-analyse the current literature on the reduction of chronic pelvic pain after botulinum toxin A (BTA) injection.

**Method:**

In July 2021 we performed a systematic search in PubMed and EMBASE to assess the benefits of BTA injection in pelvic floor muscles in women with chronic pelvic pain. Primary outcome was reduction in visual analogue scale (VAS) after treatment. Secondary outcomes evaluated were: reduction of dyspareunia, pelvic floor resting pressure and quality of life. Identified reports were assessed on quality of reporting and risk of bias. Standardized mean difference (SMD) was used to combine and analyse outcomes of the included studies.

**Results:**

Eight studies with 289 participants were considered eligible to be included in this systematic review and meta-analysis. After recalculating SMD into VAS scores (0–100), long-term follow-up (24–26 weeks) showed a significant 15-point improvement in VAS scores (95% CI: 8.8–21.5) for non-menstrual pelvic pain and a 13-point improvement (95% CI: 2.1–24.0) for dyspareunia. BTA injection had a significant effect on pelvic floor resting pressure and quality of life.

**Conclusion:**

There is limited scientific evidence on the effectiveness of BTA injections in pelvic floor muscles in women with chronic pelvic pain. The available studies show that BTA injections significantly reduce pain levels and improve quality of life at 6 months follow-up.

**Prospero ID:**

CRD42018105204.

## Introduction

Chronic pelvic pain is a common multifactorial condition affecting 6 to 27% of women aged 18–50 uearsworldwide [1]. The International Continence Society (ICS) has defined chronic pelvic pain as persistent pain lasting > 6 months or recurring episodes of abdominal-perineo-pelvic pain, hypersensitivity or discomfort often associated with elimination changes or sexual dysfunction often in the absence of organic aetiology [2].

The pathophysiology of chronic pelvic pain is not well understood. Pelvic pain can arise from structures including visceral organs, muscles and the lumbosacral nerves. Every origin has a wide variety of causes, both physical and psychological, which result in secondary muscle contraction and tenderness, known as myofascial trigger points [3]. As many as 85% of these women with CPP have dysfunction of the pelvic musculature [4]. Chronic muscle contraction is associated with a disproportionate release of acetylcholine and other neurogenic inflammatory substances from the neuromuscular junction. This abnormal acetylcholine release triggers increased muscle tension, local hypoxia and tissue distress, leading to a release of more neuropeptides: substance P, calcitonin gene-related peptide and glutamate. Together these neuropeptides activate a complex cascade resulting in a direct stimulation of peripheral nociceptors leading to pain sensation. Autonomic modulation caused by tissue ischemia and decrease of pH potentiates abnormal acetylcholine release, thereby maintaining the hypoxic environment. Central sensitisation occurs when neuropeptides lower the threshold for pain nociception and thereby maintain pain sensation, even after the primary cause of pain nociception has vanished [5, 6]. As such, hypertonic pelvic floor muscles can lead to chronic pelvic pain among other functional problems, such as urinary, sexual and defecatory problems. [7, 8].

First-line treatment of CPP consists of pelvic floor muscle stretching and treatment of myofascial triggerpoints [3]. Unfortunately, this non-invasive treatment is of limited effectiveness. A 2019 systematic review of physiotherapy interventions for CPP concluded that triggerpoint therapy might be useful in management of CPP, but the evidence is inconclusive and the quality of included studies was low [9]. When pelvic floor physical therapy fails, more invasive interventions can be proposed. A multidisciplinary approach is recommended. The injection of botulinum toxin A (BTA) may represent a reasonable second-line intervention.

An injection of BTA into the pelvic floor muscles was first described over 20 years ago [10]. BTA is a neuromuscular blocking agent, acting by binding to receptor sites on motor nerve terminals to inhibit the release of acetylcholine. When injected intramuscularly at therapeutic doses, BTA produces a localized, partial and reversible chemical denervation of the pelvic floor muscle, which results in localized muscle weakness or temporarily paralysis [11]. A full recovery of muscle strength is expected within 3 to 6 months after BTA-injection [10, 12]. In addition, BTA was found to block the release of substance P and glutamate in afferent C-fibres, leading to peripheral desensitization and, indirectly, to reduced central sensitisation [5]. In conclusion, BTA has the theoretic potential to reduce pain in chronic pelvic pain patients.

The aim of this systematic review is to investigate and to perform a meta-analysis of the available evidence regarding the efficacy of BTA injection in the pelvic floor muscles in women with chronic pelvic pain.

## Methods

### Eligibility criteria

We included studies that examined the efficacy of BTA in women with chronic pelvic pain and hypertonicity of the pelvic floor. The intervention must have been with BTA injection into the levator ani muscle.

### Literature search strategy

To identify all available studies on chronic pelvic pain and BTA published and indexed up until 14 July 2021, a systematic search strategy was applied in PubMed, Cochrane Library and EMBASE. Medical Subject Heading (MeSH) terms and Emtree terms were used in PubMed and EMBASE together with separate words or word combinations in titles and abstracts. Databases were searched with a combination of the following keywords and/or MeSH and Emtree terms: ‘pelvic’, ‘pain’, ‘botox’, ‘neuronox’, ‘botulinum toxin A’ and ‘ona-, daxi-, abo-, inco- and prabotulinumtoxin’. In addition, retrieved reviews were screened for primary studies not found in the search strategy.

### Study selection

Identification and selection of studies were conducted according to the Preferred Reporting Items for Systematic reviews and Meta-analysis (PRISMA) [13]. The automatic deduplication of the retrieved articles was performed twice with EndNote (version X8.0.1, Clarivate Analytics, Philadelphia, PA): first per searched database and then based on the relevant titles. Studies were first assessed based on title/abstract and, if convincingly irrelevant, excluded by one investigator. All remaining abstracts were screened by two investigators. Full-text publications of the selected abstracts were assessed by two investigators. Consensus on inclusion was reached in a meeting on the discrepancies. Conference abstracts were excluded if incomplete data were published.

### Data extraction

The following data were extracted from the included studies: first author, year of publication, journal of publication, study design, inclusion and exclusion criteria, number and characteristics of participants, treatment protocol, study outcome, complications and duration of follow-up. The primary outcome was non-menstrual pelvic pain and secondary outcomes were dyspareunia, the physical component of QoL and the vaginal pelvic floor resting pressure.

### Assessment of risk of bias

Different tools and checklists will be used to assess the quality of included studies depending on study design: the Strengthening the Reporting of Observational studies in Epidemiology (STROBE), the methodological index for non-randomized studies (MINORS) and the Cochrane Collaboration’s tool.

The quality of reporting in cohort studies was evaluated by using the STROBE checklist, and MINORS was used to assess the bias risk of cohort studies [14, 15]. The STROBE checklist consists of 22 items, subdivided into 34 subitems, and each subitem could take one of the following values: yes/partial/no/not applicable. Each item of the MINORS checklist could take one of the following values: 0 = not reported, 1 = reported but inadequate and 2 = reported and adequate. A study with a score ≤ 50% of the maximum score (8 for case series and cohort studies) was considered as having a ‘high risk of bias’ [16].

Risk of bias of the included RCTs was evaluated by Cochrane Collaboration’s tool [17]. Included studies were evaluated by two independent reviewers. In cases of disagreement between the reviewers, consensus was reached by discussion.

Publication bias was assessed through inspection of a funnelplot at 2 to 6 weeks after treatment for all included studies.

### Data synthesis

The standardized mean difference (SMD) was used to combine and analyse the results of the included studies. If the median and interquartile range were available, they were converted to mean and standard deviation [Higgins, 2011, 6.5.1.2]. The statistical methods used included the inverse variance method and DerSimonian-Laird estimator for tau^2^ as implemented in the R package ‘meta’ [18], which were used to assess heterogeneity between studies. Both fixed and random effects models were plotted to allow for further appraisal of the heterogeneity. All outcomes were computed (when ≥ 2 studies were involved) using R (version 3.4.4 for Windows). Outcomes were presented in one of two ways: by using rules of thumb for effect sizes or by multiplying a SMD with a typical among-person standard deviation (SD) for a particular scale [17]. We used an existing rule of thumb: < 0.3 represents a small effect, 0.3–0.8 a moderate effect and > 0.8 a large effect [17, 19]. The typical among-person standard deviation was obtained from the baseline score of one of the included studies [17]. Due to a small number of included studies, we did not use a pooled baseline standard deviation but rather chose the least favourable baseline standard deviation to prevent an overestimation of the effect. Results are presented in forest plots and plots presenting 95% confidence intervals (CIs) and means.

## Results

### Study selection

**Fig. 1:**
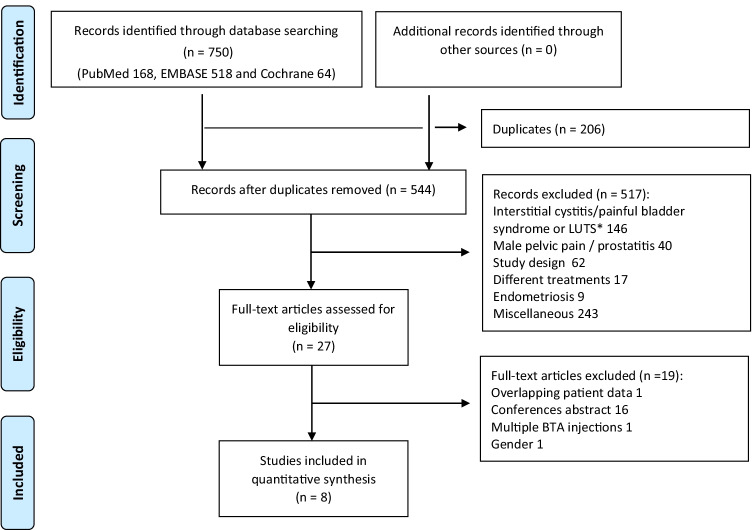
PRISMA flow diagram of the process for identification of eligible studies to be included in this systematic review and meta-analysis. *Lower urinary tract symptoms

Our search identified 750 manuscripts. Of these, eight original articles with a total of 289 participants (230 cases, 59 controls) were considered eligible to be included in this review [20–27]. The process for identification of eligible studies is presented in a PRISMA flow diagram (Fig. 1).

### Study characteristics

Main characteristics of the included studies are presented in Table 1. There were two randomized controlled trials (RCTs) [20, 22], four prospective cohort studies [24-27] and two retrospective studies (cohort and case series) [21, 23].

**Table 1:** Summary of main characteristics reported in the studies included in the systematic review and meta-analysis

First author [ref]	Study design	Number of cases	Baseline characteristics	Intervention	Outcome measures	Follow-up	STROBE	MINORS
Abbott 2006 [20]	Double-blinded, randomized, placebo- controlled trial	60 (30/30)	No significant differences, mean age I*: 30.6 ± 8.1, C**: 30.5 ± 7.5	Intervention group: 80 U BTA Control group: placebo	- VAS (dyspareunia, dysmenorrhea, dyschezia and non-menstrual pelvic pain) - Qol (EQ-5D, SF-12 and sexual activity (questionnaire scores)	0, 1, 2, 3, 4, 5 and 6 months	–	–
Adelowo 2013 [21]	Retrospective cohort study	29	Median age 55.0 (38–62), 65.5% post-menopausal	Dose: 100-300 U BTA	- Patient-reported tenderness on levator ani palpation, pain-score 0–10. - Patient-reported symptom improvement - Time to and number of repeat injections	< 6 and > 6 weeks post-treatment	22/4/2/6	8
Dessie 2019 [22]	Double-blinded, randomized, placebo- controlled trial	59 (30/29)	Difference in sexual activity, dyspareunia and faecal incontinence	Intervention group: 200 U BTA Control group: placebo	- Pain on palpation and Self-reported overall pain (VAS 0–10) - Patient global impression of severity and improvement - Pelvic Floor Distress Index (PFDI-20) - Pain Urgency Frequency score	2, 4 and 12 weeks post-treatment	–	–
Halder 2017 [23]	Retrospective case-series	50	Mean age 44.5 ± 15.2, parity 1.6 ± 1.4, 50% post-menopausal	Dose: 100-200 U BTA	- Pain score (0–10) pre- and post-treatment - Pain improvement (yes/no)	One visit in 2 to 8 weeks post treatment	19/5/4/6	8
Jarvis 2004 [24]	Prospective cohort study (pilot)	12	Mean age 31.1 (21–45), 83% endometrioses	Dose: 40 U BTA	- VAS (dyspareunia, dysmenorrhea, dyschezia and non-menstrual pelvic pain) - Qol (SF-12; EQ-5D) - Sexual activity scores - Pelvic floor muscles manometry	2, 4, 8 and 12 weeks post-treatment	15/5/7/7	9
Mooney 2021 [25]	Prospective cohort study	21	Mean age 32.9, Previous surgery for pain 13 (61.9%)	Dose: 100 U BTA	- VAS (pelvic pain, dyspareunia, dysmenorrhea, dyschezia and dysuria) - World Health organization Quality of life - Pain catastrophizing scale - Pelvic floor dysfunction score and pelvic muscle map score	6, 12 and 26 weeks post-treatment	19/3/5/7	10
Morrissey 2015 [26]	Prospective pilot open-label study	21	Mean age 35.1 (22–50), 72% sexual active	Dose: Up to 300 U BTA injection: using EMG guidance	- Global Respons Assessment (GRA scale) for pelvic pain - VAS scores for pain and dyspareunia - QoL and sexual function - Digital examination of pelvic floor tone and tenderness - vaginal manometry	4, 8, 12 and 24 weeks post- treatment	18/5/4/7	11
Nesbitt 2012 [27]	Prospective cohort study	37	Mean age 30 (26–41)	Dose: 100 U BTA Group 1: 1 injection Group 2: 2 or more injections	- VAS (dyspareunia, dysmenorrhea, dyschezia and non-menstrual pelvic pain) - vaginal manometry	4, 12 and 26 weeks post-treatment	17/8/5/4	11

Baseline characteristics: values are presented as median (interquartile range) or mean ± standard deviation

STROBE: the sum of each of the values (yes/partial/no/not applicable) is presented with a joint maximum score of 34

MINORS: the numbers presented are the sum of the values (0 = not reported, 1 = reported but inadequate and 2 = reported and adequate) with a maximum total score of 16

*Intervention group, **control group, *-not applicable

The treatment protocol ranged from 40 U BTA to a maximum of 300 U BTA injection in the pelvic floor muscles. All studies assessed pelvic pain and/or dyspareunia using a pain rating scale, i.e. the visual analogue scale (VAS) 0–10 [22, 23, 25], VAS 0–100 [20, 24, 27] and a pain score ranging from 0 to 10 [21, 23]. One study did not specify the results (i.e. no standard deviation, interquartile range or *p* value), and at email inquiry the author was not able to share data beyond what was published [24]. Published data of two other papers were incomplete, but after email inquiry, the authors were not able to share missing data [23, 26]. Hence, depending on the outcome parameter, studies were included in the meta-analysis for pelvic pain (6 studies) and dyspareunia (3 studies). Quality of life (QoL) was assessed by the Short Form 12 physical health composite survey (SF-12) in three studies [20, 24, 26]. Four studies measured the vaginal pelvic floor resting pressure by vaginal manometry [20, 24, 26, 27].

**Fig. 2:**
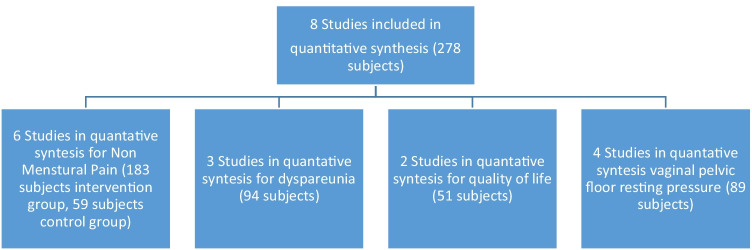
Flow diagram: number of included studies and subjects in the meta-analysis

Data from a control group with women with non-menstrual pelvic pain were extracted from both RCTs [20, 22]. Results are presented in Table 2 and number of included studies and subjects in the meta-analysis is presented in Fig. 2.

**Table 2:** Primary and secondary outcomes of the studies included in the systematic review and meta-analysis

First author [ref]	NMPP Baseline	NMPP 2–6 weeks FU	NMPP 12 weeks FU	NMPP 24–26 weeks FU	Dyspareunia Baseline	Dyspareunia 2–6 weeks FU	Dyspareunia 12 weeks FU	Dyspareunia 24–26 weeks FU	QoL Baseline	QoL 12 weeks FU	VPFRP Baseline	VPFRP 12 weeks FU
**Intervention group**												
Abbott 2006 [20]	51 (24–70)	23 (0–55)	25 (0–56)	27 (1–50)	66 (49–90)	18 (0–66)	14 (0–58)	44 (3–60)	38.44 (31.65–46.64)	43.94 (33.44–54.09)	49 (43–56)	34 (26–36)
Adelowo 2013 [21]	9.5 (8–10)	0.0 (0–3)	–	–	–	–	–	–	–	–	–	–
Desssie 2019 [22]	7 (6–10)	6 (2–7)	6 (3–7)	–	–	–	–	–	–	–	–	–
Halder 2017 [23]	6.4 ± 1.8	3.7 ± 4	–	–	–	–	–	–	–	–	–	–
Jarvis 2004 [24]	N.D.A.	N.D.A.	N.D.A.	–	–	–	–	–	N.D.A	N.D.A.	57* (29-82)	43* (30-57)
Mooney 2021 [25]	8 (6–9)	7 (2–8)	6 (2–8)	6 (4–9)	10 (8–10)	6 (3–10)	8 (4–10)	10 (4–10)	–	–	–	–
Morrissey 2015 [26]	4.3	N.D.A.	N.D.A.	3.2 (*p* = 0.01)	7.8	6.7 (*p* = 0.059)	5.6 (*p* = 0.011)	5.4 (*p* = 0.004)	40.0	43.1 (p = <0.05)	44.7	37.3 (p = <0.05)
Nesbitt 2012 [27]	45 (25–51)	38 (7–52)	15 (0–47)	20 (0–36)	54 (12–76)	33 (5–70)	25 (0–54)	30 (5–46)	–	–	47 (38–54)	36 (32–39)
**Control group**												
Abbott 2006 [20]	4,7 (3,3-6,2)	4 (3,5-6,7)	4 (2,4–6)	4 (1,1-6,5)	–	–	–	–	–	–	–	–
Desssie 2019 [22]	6,1 (6–10)	6,8 (6–7,8)	6,1 (2,1-7,1)	–	–	–	–	–	–	–	–	–

NMPP = non-menstrual pelvic pain, QoL = quality of life, physical health composite scale; VPFRP = vaginal pelvic floor resting pressure; FU = follow-up

Values are presented as median (interquartile range), mean ± standard deviation or *p* value

NDA = no data available

*Mean (range)

### Risk of bias of included studies

**Fig. 3:**
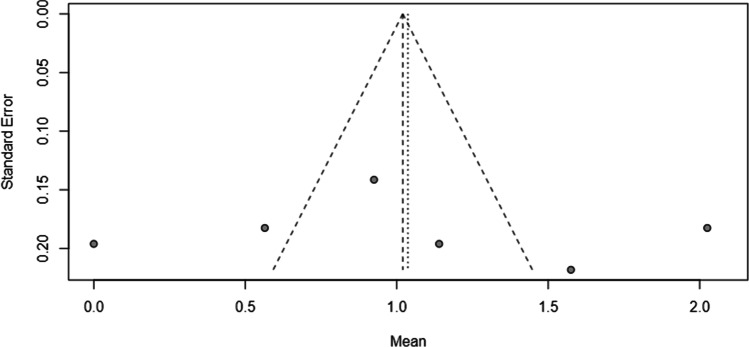
Funnelplot for assessment of publication bias in studies evaluating BTA treatment in women with chronic pelvic pain

The STROBE checklist [14] revealed wide differences in the reporting of items. The complete reporting of items ranged from 44% [24] to 65% [21]. Items addressing bias, missing data, confounders, limitations and generalisability were reported in < 50% of the included studies. The included RCT was classified as a low risk of bias. Risk of bias in cohort studies assessed by the MINORS checklist [15] revealed a high risk of bias in two out of six studies [21, 23]. A summary of results is presented in Table 1. Figure 3 shows minimal evidence of publication bias.

### Synthesis of results

#### Primary outcomes

**Fig. 4:**
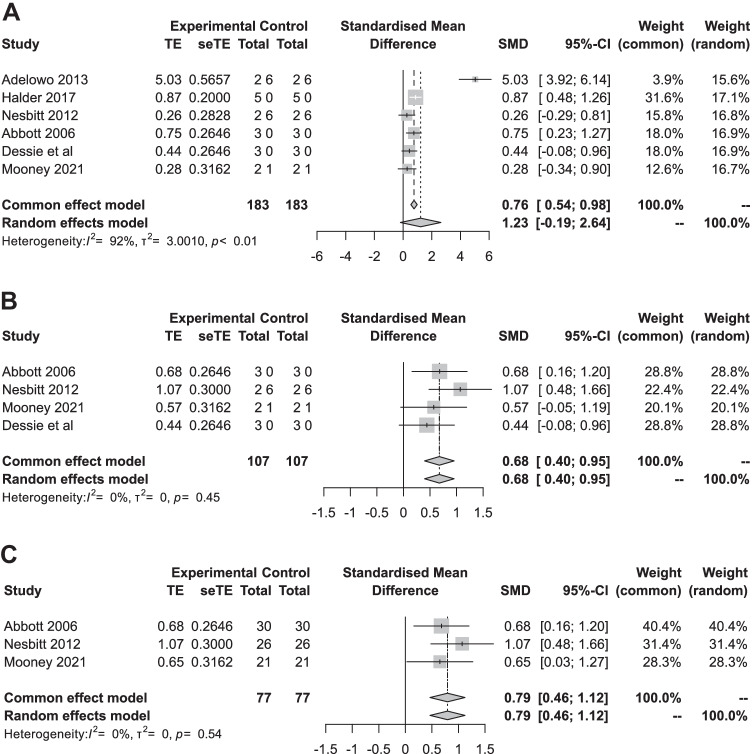
Meta-analysis of non-menstrual pelvic pain. Forest plots of standardized mean differences between mean values before the BTA injection and 2–6 (A), 12 (B) and 24–26 (C) weeks after BTA injection for the primary outcome: non-menstrual pelvic pain assessed by VAS scores (0–10 or 0–100) or pain score (0–10)

**Fig. 5:**
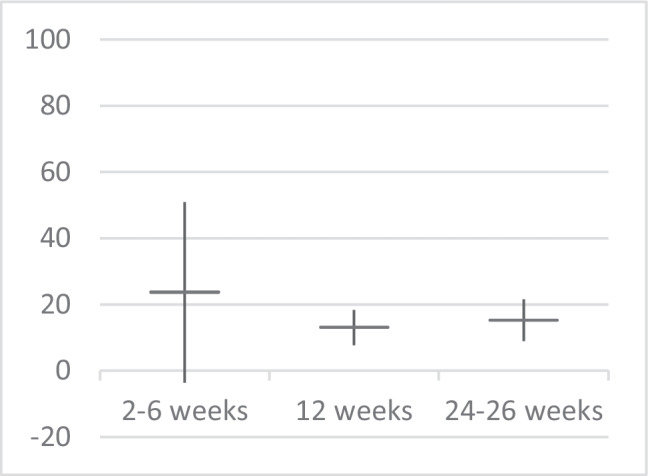
Non-menstrual pelvic pain; improvement of VAS 0–100 compared to baseline. Calculation: random effects model SMD × 19.26 (baseline standard deviation Nesbitt et al.)

Forest plots of the meta-analyses of the primary outcome non-menstrual pelvic pain are presented in Fig. 4. Non-menstrual pelvic pain decreased significantly at 12 weeks (SMD 0.68; 95% CI: 0.40–0.95; I^2^ = 0%) and 24–26 weeks follow-up (SMD 0.79; 95% CI: 0.46–1.12; I^2^ = 0%). The most substantial decrease in VAS score was found in the period of 2 to 6 weeks follow-up (SMD 1.23; 95% CI: −0.19–2.64; I^2^ = 92%), but this difference was not statistically significant (Fig. 4). Recalculating SMD to VAS scores (0–100) using the baseline standard deviation of Nesbitt et al. (SD baseline 19.26) resulted in a mean decrease in VAS score of 24 points (95% CI: −3.6–50.8) at 2 to 6 weeks, 13 points (95% CI: 7.7–18.3) after 12 weeks and 15 points (95% CI: 8.8–21.5) after 26 weeks of follow-up compared to baseline (Fig. 5).

The control group did not show a significant change at 2 to 6 weeks follow-up (SMD −0.12; 95% CL: −0.95-0.72) and 12 weeks follow-up (SMD 0.14; 95% CL: −0.22-0.51). Furthermore, the effect in the control group was smaller compared to the intervention group (12 weeks follow-up: intervention group SMD 0.68 vs. control group SMD 0.14).

#### Secondary outcomes

Dyspareunia VAS scores decreased considerably at 2 to 6 weeks follow-up (SMD 0.88; 95% CI: 0.41–1.35; I^2^ = 47%) and were maintained at 12 weeks (SMD 0.90; 95% CI: 0.39–1.41; I^2^ = 58%) and 24–26 weeks follow-up (SMD 0.43; 95% CI: 0.07–0.79; I^2^ = 24%). Re-expressing SMD to VAS scores (0–100) using the Abbott et al. baseline standard deviation (SD baseline 30.37) resulted in a mean decrease in VAS scores of 27 points (95% CI: 12.5–41.0) at 2 to 6 weeks, 27 points (95% CI: 11.8–42.8) at 12 weeks and 13 points (95% CI: 2.1–24.0) at 24–26 weeks compared to baseline.

The physical component of QoL increased statistically significantly compared to baseline after 12 weeks follow-up. The standardized mean difference was −0.50 (95% CI: −0.90 to −0.11), showing a moderate positive effect of BTA injection on the QoL physical health composite scale. The vaginal pelvic floor resting pressure decreased statistically significantly at 12 weeks follow-up, with a large effect rating of 1.05 SMD (95% CI: 0.48–1.6).

## Discussion

### Main findings

We performed a systematic review and meta-analysis of the available evidence on botulinum toxin A injection as a treatment for the management of chronic pelvic pain. Non-menstrual pelvic pain showed a significant improvement at long-term follow-up (12–26 weeks after BTA injection). Improvement was also seen at short-term follow-up (2 to 6 weeks after BTA injection), although this difference was not statistically significant. Dyspareunia also improved at short- and long-term follow-up. In addition, a decrease of vaginal pelvic floor resting pressure and an increase of QoL was seen. However, the quality of included studies is low and the clinical relevance of BTA remains largely unclear.

### Interpretation

Although these results might seem promising, the included RCTs by Abbott et al. and Dessie et al. did not find a statistically significant intergroup difference for individual pain scores after BTA or placebo injection [20, 22]. In both trials, women were not required to have had pelvic floor physical therapy prior to inclusion in the study. Pelvic floor physical therapy was part of the treatment protocol by Dessie et al. [22]. Moreover, the repeated measurement of the pelvic floor pressure by perineometry during the trial is also part of the standard physical therapy treatment for patients with pelvic pain [20]. Therefore, through repeated pelvic floor measurements, patients could have had pelvic floor physical therapy training during follow-up. In addition, muscle needling is associated with a decrease in frequency of muscle spasms [28], which possibly allows the saline injection to give a decrease in pelvic pain. These factors may have resulted in the absence of a significant intergroup difference.

A recent retrospective study by Jha et al. assessed the impact (PGI-I) of BTA treatment. This paper demonstrated improvement (very much better and much better) in symptoms in 72% of women after one or multiple BTA injections [29]. However, no additional analysis was performed to differentiate between one or multiple injections.

Studies evaluating the efficacy of BTA treatment in women with provoked vulvodynia showed contrasting results. Provoked vulvodynia is a chronic discomfort in the vulva without objective findings of specific signs of neurological, infectious or anatomical disorders resulting in the hypertonicity of the superficial muscles of the perineum [30]. Pelletier et al. injected 100 U BTA, using EMG guidance, in the superficial pelvic floor muscles of 20 women with vulvodynia resulting in a statistically significant pain reduction of 3.52 points (*p* < 0.001) in VAS scores (0–10) at 6 months after injection. However, Diomande et al.’s [31] RCT including 32 patients compared three treatment arms: a single injection of 50 units of BTA (arm A), of 100 units of BTA (arm B) and of a placebo (arm C). Three months after initial injection, no significant differences in pain were observed among the study arms (cotton swab-provoked VAS, *p* = 0.857), and no significant intragroup improvements occurred (arm A: *p* = 0.41; arm B: *p* = 0.239; arm C: *p* = 0.623). The study results could be explained by a difference in pain localisation and placement of injections due to the absence of EMG guidance in the RCT. In addition, similar to patients in the study by Abbott et al., patients in this study were not referred to undergo pelvic floor muscle training prior to the study.

A Dutch cohort study included 50 patients with dyspareunia or vaginismus and pelvic floor hypertonicity already treated by a physical therapist and/or sexologist/psychologist [31, 32]. The included patients received 50 IU BTA administered into the puborectalis muscle under EMG guidance. After 47 months follow-up time, 45 patients (87%) had no signs of hypertonicity, and 37 patients did not experience dyspareunia after a single BTA injection. These results support our findings and endorse the demand for more research on EMG-guided BTA injections in the pelvic floor.

Another systematic review on the topic by Meister et al. [33] was conducted 2 years prior to the present study. The results of that review are largely similar but might overestimate the effect of BTA. In the present review we did a thorough assessment of the quality of the included studies and the risk of bias by applying the MINORS and STROBE checklist. We regard it as important to emphasize the low quality of the current literature.

### Strengths and limitations

This study has several limitations that should be acknowledged. First, the current literature lacks RCTs, well-designed case-controlled studies and consistency in dosing and method of injection. More well-designed studies are needed on the topic before recommending regular injections of BTA in clinical practice.

Second, the majority of included studies were case-series studies, thereby making a placebo effect of treatment a possibility. Finally, we could not retrieve the complete data required for meta-analysis in all publications, and several authors were not able to share the missing data [23, 24, 26]. Despite the missing data, a statistically significant and potentially clinically relevant result was seen in our meta-analysis.

Third, heterogeneity in the included studies was seen (NMPP at 2 to 6 weeks follow-up; I^2^: 92%, *p* < 0.01 and VPFRP at 12 weeks FU; I^2^: 68%, *p* < 0.03) because of different inclusion criteria and patient populations. Adelowo et al. and Halder et al. showed an evidently higher mean age. Furthermore, the study presented an evidently higher baseline pain score compared to the pain score in other populations. Jarvis et al. included 12 patients of whom 10 had been previously diagnosed with endometriosis. Nevertheless, both the fixed effect model and the random effect model showed a significant positive effect. Only the quantity of improvement might differ between different patient populations. There might be a role for pre-treatment pelvic floor physiotherapy and EMG-guided injections, but the samples were too small to firmly conclude on this.

Fourth, outcomes could be influenced by a regression to the mean. We analysed repeated measurements on the same subject, which ensures that random error, or random fluctuations in a subject, might occur [34]. Unfortunately, this could not be tested in our analyses. Additionally, we used the least favourable baseline standard deviation of the included studies to re-express SMD back to the original measurement, thereby possibly leading to an underestimation of the improvement of the original measurement. Baseline standard deviation is expected to be the best representative for the study population [17]. However, despite using the least favourable baseline standard deviation, we found an evident improvement in symptoms.

### Conclusion

This meta-analysis shows that BTA treatment reduces pain levels and improves quality of life in women with chronic pelvic pain. To date, there is however a scarcity of well-designed placebo-controlled studies with clear inclusion and exclusion criteria, standard therapeutic treatment protocols, appropriate duration of follow-up and blinded outcome evaluations. We expect a role for pre-treatment pelvic floor physiotherapy and EMG-guided injections, but the samples were too small to firmly conclude on this.

Due to the low quality of the available studies, no firm conclusions on effectiveness can be made and the clinical relevance of BTA remains largely unclear. More research is needed before recommending BTA treatment in clinical practices for women with chronic pelvic pain. Future studies need to provide more details to assess relevant issues, widely ranging from the population to additional pelvic floor training and treatment protocols.
